# Supplementary material for: Residual Interactions of LL-37 with POPC and POPE:POPG Bilayer Model Studied by All-Atom Molecular Dynamics Simulation
Source: Int J Mol Sci. 2022 Nov 2;23(21):13413. doi: 10.3390/ijms232113413 (PMC9654553; doi:10.3390/ijms232113413)
Supplement: Supplementary file 1 [file ijms-23-13413-s001.zip › ijms-1860597-supplementary.pdf]

SUPPLEMENTARY DATA

Table S1. The residual interactions of LL-37 with POPC membrane.

No.	Interaction	Distance	Category	From	To
1	:LYS8 - :PC426	5.07	Hydrophobic	:LYS8	:PC426
2	:PA134:C13 - :LEU2	5.06	Hydrophobic	:PA134:C13	:LEU2
3	:OL136 - :LEU2	4.27	Hydrophobic	:OL136	:LEU2
4	:OL136 - :LEU2	4.86	Hydrophobic	:OL136	:LEU2
5	:OL136 - :LEU2	5.11	Hydrophobic	:OL136	:LEU2
6	:OL412 - :LEU2	5.21	Hydrophobic	:OL412	:LEU2
7	:OL136 - :LEU1	5.09	Hydrophobic	:OL136	:LEU1
8	:OL136:C117 - :LEU1	5.08	Hydrophobic	:OL136:C117	:LEU1
9	:OL412:C13 - :ARG7	4.87	Hydrophobic	:OL412:C13	:ARG7
10	:PHE6 - :PC426	4.52	Hydrophobic	:PHE6	:PC426
11	:PHE6 - :PA134:C13	5.21	Hydrophobic	:PHE6	:PA134:C13
12	:PHE5 - :OL454:C13	4.93	Hydrophobic	:PHE5	:OL454:C13
13	:PHE5 - :OL454	4.22	Hydrophobic	:PHE5	:OL454
14	:PHE5 - :OL454	5.04	Hydrophobic	:PHE5	:OL454
15	:PHE6 - :OL340	5.47	Hydrophobic	:PHE6	:OL340
16	:PHE6 - :OL277	4.63	Hydrophobic	:PHE6	:OL277
**No.**	**Interaction**	**Distance**	**Category**	**From**	**To**
1	:ARG7:HH22 - :PC60:O33	2.00	Hydrogen Bond	:ARG7:HH22	:PC60:O33
2	:ARG7:HH22 - :PC60:O33	2.00	Hydrogen Bond	:ARG7:HH22	:PC60:O33
3	:LYS10:HZ2 - :PC453:O33	3.10	Hydrogen Bond	:LYS10:HZ2	:PC453:O33
4	:LYS10:HZ2 - :PC453:O33	3.10	Hydrogen Bond	:LYS10:HZ2	:PC453:O33
5	:ARG7:HH11 - :PC453:O22	1.75	Hydrogen Bond	:ARG7:HH11	:PC453:O22
6	:ARG7:HH11 - :PC453:O22	1.75	Hydrogen Bond	:ARG7:HH11	:PC453:O22
7	:ARG7:HH21 - :PC411:O34	1.70	Hydrogen Bond	:ARG7:HH21	:PC411:O34
8	:ARG7:HH21 - :PC411:O34	1.70	Hydrogen Bond	:ARG7:HH21	:PC411:O34
9	:LYS8:HZ1 - :PC396:O33	1.87	Hydrogen Bond	:LYS8:HZ1	:PC396:O33
10	:LYS8:HZ1 - :PC396:O33	1.87	Hydrogen Bond	:LYS8:HZ1	:PC396:O33
11	:SER9:HG - :PC276:O33	1.59	Hydrogen Bond	:SER9:HG	:PC276:O33
12	:SER9:HG - :PC276:O33	1.59	Hydrogen Bond	:SER9:HG	:PC276:O33
13	:PHE5:HN - :PC276:O22	2.56	Hydrogen Bond	:PHE5:HN	:PC276:O22
14	:PHE6:HN - :PC276:O22	2.04	Hydrogen Bond	:PHE6:HN	:PC276:O22
15	:PHE5:HN - :PC276:O22	2.56	Hydrogen Bond	:PHE5:HN	:PC276:O22
16	:PHE6:HN - :PC276:O22	2.04	Hydrogen Bond	:PHE6:HN	:PC276:O22
17	:LEU1:HT2 - :PC210:O22	2.85	Hydrogen Bond	:LEU1:HT2	:PC210:O22
18	:LEU1:HT2 - :PC210:O22	2.85	Hydrogen Bond	:LEU1:HT2	:PC210:O22
19	:LEU1:HT1 - :PC135:O34	1.99	Hydrogen Bond	:LEU1:HT1	:PC135:O34
20	:LEU1:HT1 - :PC135:O34	1.99	Hydrogen Bond	:LEU1:HT1	:PC135:O34
21	:LYS10:HZ1 - :PC60:O34	3.25	Hydrogen Bond;Electrostatic	:LYS10:HZ1	:PC60:O34
22	:LYS10:HZ3 - :PC453:O34	1.68	Hydrogen Bond;Electrostatic	:LYS10:HZ3	:PC453:O34
23	:LYS8:HZ3 - :PC411:O33	1.75	Hydrogen Bond;Electrostatic	:LYS8:HZ3	:PC411:O33
24	:LYS10:HZ1 - :PC60:O34	3.25	Hydrogen Bond;Electrostatic	:LYS10:HZ1	:PC60:O34
25	:LYS10:HZ3 - :PC453:O34	1.68	Hydrogen Bond;Electrostatic	:LYS10:HZ3	:PC453:O34
26	:LYS8:HZ3 - :PC411:O33	1.75	Hydrogen Bond;Electrostatic	:LYS8:HZ3	:PC411:O33
27	:ARG7:NH1 - :PC60:O34	2.94	Electrostatic	:ARG7:NH1	:PC60:O34

28	:ARG7:NH1 - :PC453:O34	5.46	Electrostatic	:ARG7:NH1	:PC453:O34
29	:LYS12:NZ - :PC426:O33	5.01	Electrostatic	:LYS12:NZ	:PC426:O33
30	:ARG7:NH2 - :PC411:O33	4.73	Electrostatic	:ARG7:NH2	:PC411:O33
31	:LYS8:NZ - :PC396:O34	5.07	Electrostatic	:LYS8:NZ	:PC396:O34
32	:LEU1:N - :PC135:O33	5.28	Electrostatic	:LEU1:N	:PC135:O33
33	:PC135:N31 - :ASP4:OD2	3.70	Electrostatic	:PC135:N31	:ASP4:OD2

Table S2. The residual interactions of LL-37 with POPE:POPG membrane.

No.	Interaction	Distance	Category	From	To
1	:PA347:C13 - :VAL21	3.78	Hydrophobic	:PA347:C13	:VAL21
2	:PA131:C116 - :PRO33	4.71	Hydrophobic	:PA131:C116	:PRO33
3	:PA488:C116 - :PRO33	4.65	Hydrophobic	:PA488:C116	:PRO33
4	:ARG7 - :PGR537	5.42	Hydrophobic	:ARG7	:PGR537
5	:ARG29 - :PGR489	5.41	Hydrophobic	:ARG29	:PGR489
6	:LYS25 - :PGR489	4.44	Hydrophobic	:LYS25	:PGR489
7	:LYS12 - :PE72	5.17	Hydrophobic	:LYS12	:PE72
8	:PRO33 - :PA488	5.31	Hydrophobic	:PRO33	:PA488
9	:PRO33 - :PA374	4.71	Hydrophobic	:PRO33	:PA374
10	:PRO33 - :PA131	5.04	Hydrophobic	:PRO33	:PA131
11	:PRO33 - :OL61	4.23	Hydrophobic	:PRO33	:OL61
12	:ARG29 - :OL511	4.67	Hydrophobic	:ARG29	:OL511
13	:VAL32 - :OL511	4.51	Hydrophobic	:VAL32	:OL511
14	:VAL32 - :OL511	4.35	Hydrophobic	:VAL32	:OL511
15	:VAL32 - :OL511	4.74	Hydrophobic	:VAL32	:OL511
16	:LYS10 - :OL121	5.23	Hydrophobic	:LYS10	:OL121
17	:OL121:C13 - :LYS10	4.22	Hydrophobic	:OL121:C13	:LYS10
18	:PA488 - :LEU31	5.17	Hydrophobic	:PA488	:LEU31
19	:PA488 - :LEU31	5.16	Hydrophobic	:PA488	:LEU31
20	:PA38 - :LEU28	4.91	Hydrophobic	:PA38	:LEU28
21	:PA488:C13 - :LEU28	4.45	Hydrophobic	:PA488:C13	:LEU28
22	:PGR489 - :LEU28	5.47	Hydrophobic	:PGR489	:LEU28
23	:OL382 - :LEU2	5.17	Hydrophobic	:OL382	:LEU2
24	:OL538:C13 - :LEU2	4.36	Hydrophobic	:OL538:C13	:LEU2
25	:OL382 - :LEU1	4.57	Hydrophobic	:OL382	:LEU1
26	:OL382 - :LEU1	5.23	Hydrophobic	:OL382	:LEU1
27	:PA146:C13 - :LEU1	4.92	Hydrophobic	:PA146:C13	:LEU1
28	:PE252 - :LEU1	4.62	Hydrophobic	:PE252	:LEU1
29	:PA38 - :ILE24	5.33	Hydrophobic	:PA38	:ILE24
30	:PA38:C13 - :ILE24	4.94	Hydrophobic	:PA38:C13	:ILE24
31	:PA71:C116 - :ILE24	4.79	Hydrophobic	:PA71:C116	:ILE24
32	:PA71 - :ILE20	5.33	Hydrophobic	:PA71	:ILE20
33	:OL121 - :ILE13	5.36	Hydrophobic	:OL121	:ILE13
34	:PGR402 - :ILE13	5.47	Hydrophobic	:PGR402	:ILE13
35	:PA509:C13 - :ARG34	3.92	Hydrophobic	:PA509:C13	:ARG34
36	:PA38:C13 - :ARG23	4.32	Hydrophobic	:PA38:C13	:ARG23
37	:PHE17 - :PA71	5.17	Hydrophobic	:PHE17	:PA71
38	:PHE6 - :PA536:C13	4.19	Hydrophobic	:PHE6	:PA536:C13
39	:PHE17 - :PA401:C13	3.73	Hydrophobic	:PHE17	:PA401:C13
40	:PHE5 - :PA380:C13	4.50	Hydrophobic	:PHE5	:PA380:C13
41	:PHE27 - :PA38	4.45	Hydrophobic	:PHE27	:PA38

42	:PHE17 - :PA347:C13	5.22	Hydrophobic	:PHE17	:PA347:C13
43	:PHE17 - :PA347	5.48	Hydrophobic	:PHE17	:PA347
44	:PHE27 - :PA233	5.17	Hydrophobic	:PHE27	:PA233
45	:PHE6 - :OL538	5.04	Hydrophobic	:PHE6	:OL538
46	:PHE5 - :OL382:C13	4.98	Hydrophobic	:PHE5	:OL382:C13
47	:PHE5 - :OL382	4.21	Hydrophobic	:PHE5	:OL382
48	:PHE6 - :OL382	5.31	Hydrophobic	:PHE6	:OL382

No.	Interaction	Distance	Category	From	To
1	:PGR381:HO6A - :SER9:OG	2.17	Hydrogen Bond	:PGR381:HO6A	:SER9:OG
2	:ARG7:HH11 - :PGR537:O34	2.16	Hydrogen Bond	:ARG7:HH11	:PGR537:O34
3	:THR35:HN - :PGR510:O22	2.50	Hydrogen Bond	:THR35:HN	:PGR510:O22
4	:ARG34:HE - :PGR510:O12	1.85	Hydrogen Bond	:ARG34:HE	:PGR510:O12
5	:ARG34:HH21 - :PGR510:O12	2.84	Hydrogen Bond	:ARG34:HH21	:PGR510:O12
6	:LYS25:HZ2 - :PGR489:O36	2.65	Hydrogen Bond	:LYS25:HZ2	:PGR489:O36
7	:LYS25:HZ3 - :PGR489:O35	1.62	Hydrogen Bond	:LYS25:HZ3	:PGR489:O35
8	:ARG29:HE - :PGR489:O33	1.83	Hydrogen Bond	:ARG29:HE	:PGR489:O33
9	:LEU1:HT3 - :PGR411:O36	2.11	Hydrogen Bond	:LEU1:HT3	:PGR411:O36
10	:LEU1:HT2 - :PGR411:O35	1.64	Hydrogen Bond	:LEU1:HT2	:PGR411:O35
11	:LYS12:HZ1 - :PGR381:O33	1.93	Hydrogen Bond	:LYS12:HZ1	:PGR381:O33
12	:LYS8:HZ1 - :PGR381:O33	2.76	Hydrogen Bond	:LYS8:HZ1	:PGR381:O33
13	:LYS8:HZ1 - :PGR381:O31	2.74	Hydrogen Bond	:LYS8:HZ1	:PGR381:O31
14	:LYS8:HZ1 - :PGR381:O22	2.17	Hydrogen Bond	:LYS8:HZ1	:PGR381:O22
15	:LYS8:HZ3 - :PGR381:O22	2.00	Hydrogen Bond	:LYS8:HZ3	:PGR381:O22
16	:LYS15:HZ2 - :PE72:O33	1.80	Hydrogen Bond	:LYS15:HZ2	:PE72:O33
17	:ASN30:HD21 - :PE60:O33	1.94	Hydrogen Bond	:ASN30:HD21	:PE60:O33
18	:ARG19:HH22 - :PE39:O34	1.71	Hydrogen Bond	:ARG19:HH22	:PE39:O34
19	:ARG23:HH11 - :PE39:O12	1.87	Hydrogen Bond	:ARG23:HH11	:PE39:O12
20	:LYS8:HZ2 - :PE252:O33	1.81	Hydrogen Bond	:LYS8:HZ2	:PE252:O33
21	:ARG23:HE - :PE234:O34	2.90	Hydrogen Bond	:ARG23:HE	:PE234:O34
22	:LYS10:HZ1 - :PE120:O31	2.56	Hydrogen Bond	:LYS10:HZ1	:PE120:O31
23	:LYS10:HZ1 - :PE120:O22	1.97	Hydrogen Bond	:LYS10:HZ1	:PE120:O22
24	:ARG29:HH21 - :PGR489:O33	1.99	Hydrogen Bond;Electrostatic	:ARG29:HH21	:PGR489:O33
25	:LYS12:HZ2 - :PE72:O34	1.60	Hydrogen Bond;Electrostatic	:LYS12:HZ2	:PE72:O34
26	:ARG19:HH12 - :PE39:O33	1.72	Hydrogen Bond;Electrostatic	:ARG19:HH12	:PE39:O33
27	:ARG23:HH12 - :PE39:O33	2.08	Hydrogen Bond;Electrostatic	:ARG23:HH12	:PE39:O33
28	:ARG23:HH22 - :PE39:O33	1.71	Hydrogen Bond;Electrostatic	:ARG23:HH22	:PE39:O33
29	:ARG34:HH12 - :PE255:O34	2.37	Hydrogen Bond;Electrostatic	:ARG34:HH12	:PE255:O34
30	:ARG34:HH22 - :PE255:O34	2.05	Hydrogen Bond;Electrostatic	:ARG34:HH22	:PE255:O34
31	:ARG23:HH21 - :PE234:O34	1.87	Hydrogen Bond;Electrostatic	:ARG23:HH21	:PE234:O34
32	:LYS10:HZ3 - :PE120:O33	1.81	Hydrogen Bond;Electrostatic	:LYS10:HZ3	:PE120:O33
33	:PE153:HN1C - :GLU16:OE1	1.77	Hydrogen Bond;Electrostatic	:PE153:HN1C	:GLU16:OE1
34	:ARG7:NH1 - :PGR537:O33	4.81	Electrostatic	:ARG7:NH1	:PGR537:O33
35	:ARG34:NH2 - :PGR510:O33	4.48	Electrostatic	:ARG34:NH2	:PGR510:O33
36	:LYS10:NZ - :PGR450:O33	4.85	Electrostatic	:LYS10:NZ	:PGR450:O33
37	:LYS12:NZ - :PGR381:O34	4.30	Electrostatic	:LYS12:NZ	:PGR381:O34
38	:LYS8:NZ - :PGR381:O34	5.15	Electrostatic	:LYS8:NZ	:PGR381:O34
39	:LYS15:NZ - :PE72:O34	5.21	Electrostatic	:LYS15:NZ	:PE72:O34
40	:LYS8:NZ - :PE252:O34	5.19	Electrostatic	:LYS8:NZ	:PE252:O34
41	:ARG19:NH1 - :PE234:O34	5.54	Electrostatic	:ARG19:NH1	:PE234:O34
